# Supplementary material for: Plasma Functionalization of Multi-Walled Carbon Nanotubes for Ammonia Gas Sensors
Source: Materials (Basel). 2022 Oct 18;15(20):7262. doi: 10.3390/ma15207262 (PMC9607432; doi:10.3390/ma15207262)
Supplement: Supplementary file 1 [file materials-15-07262-s001.zip › materials-1962697-supplementary.pdf]

materials

Article

Plasma Functionalization of Multi-Walled Carbon Nanotubes for Ammonia Gas Sensors

Alexander G. Bannov [1,*], Anton M. Manakhov [2,3,*] and Dmitry V. Shtansky [2]

1 Department of Chemistry and Chemical Engineering, Novosibirsk State Technical University, 20 K. Marx, 630073 Novosibirsk, Russia
2 National University of Science and Technology MISIS, Leninsky Prospekt 4, 119049 Moscow, Russia
3 Research Institute of Clinical and Experimental Lymphology—Branch of the Institute of Cytology and Genetics, Siberian Branch of Russian Academy of Sciences, 2 Timakova st., 630060 Novosibirsk, Russia
* Correspondence: bannov.alexander@gmail.com (A.G.B.); ant-manahov@ya.ru (A.M.M.)

Table S1. Response time of plasma functionalized MWCNT-based gas sensors.

Duration of treatment	Response time, s		
	100 ppm	250 ppm	500 ppm
Non-treated sample	390	407	349
2 min	399	409	358
5 min	377	432	484
7 min	385	336	333

Citation: Bannov, A.G.; Manakhov, A.M.; Shtansky, D.V. Plasma Functionalization of Multi-Walled Carbon Nanotubes for Ammonia Gas Sensors. *Materials* **2022**, *15*, 7262. https://doi.org/10.3390/ma15207262

Academic Editor: Cai Shen

Received: 25 September 2022
Accepted: 16 October 2022
Published: 18 October 2022

Publisher's Note: MDPI stays neutral with regard to jurisdictional claims in published maps and institutional affiliations.

Copyright: © 2022 by the authors. Licensee MDPI, Basel, Switzerland. This article is an open access article distributed under the terms and conditions of the Creative Commons Attribution (CC BY) license (https://creativecommons.org/licenses/by/4.0/).
